# Higher one-year achievement rate of serum phosphate associated with lower cardiovascular mortality in hemodialysis patients

**DOI:** 10.1186/s12882-021-02547-z

**Published:** 2021-12-01

**Authors:** Weichen Zhang, Guoxin Ye, Zhaori Bi, Weisheng Chen, Jing Qian, Minmin Zhang, Ding Ding, Mengjing Wang, Jing Chen

**Affiliations:** 1 Division of Nephrology, Huashan Hospital, Fudan University, 12 Middle Wurumuqi Road, Shanghai, 200040 China; 2 National Clinical Research Center for Aging and Medicine, Huashan Hospital, Fudan University, 12 Middle Wurumuqi Road, Shanghai, 200040 China; 3 Institute of Neurology, Huashan Hospital, Fudan University, Shanghai, China

**Keywords:** Achievement rate of serum phosphate, Cardiovascular mortality, Relative weights, Mineral and bone metabolism disorders

## Abstract

**Background:**

Estimation of phosphate load in hemodialysis patients is always controversial in clinical practice. The aim of this study was to verify individual achievement rate of serum phosphate as the evaluation of phosphate load through investigating its impact on cardiovascular mortality in hemodialysis patients.

**Methods:**

This was a single-center, retrospective cohort study. A total of 251 maintenance hemodialysis patients were enrolled. The individual achievement rate of serum phosphate was defined as the times of tests within the target range divided by total times of tests over a period of time. Cox regression model was used to examine the relationship between individual achievement rate of serum phosphate and cardiovascular mortality.

**Results:**

The mean age of the study population was 61 ± 13 years old. A total of 44 (17.5%) patients died from cardiovascular disease (CVD) during a median follow-up of 65 months. Multivariable Cox analysis showed that one-year serum phosphate achievement rate of 0% (HR = 4.117, *P* = 0.016) and 25% (HR = 3.343, *P* = 0.023) increased the risk of cardiovascular mortality while the achievement rate of 50% (HR = 2.129, *P* = 0.162) and 75% (HR = 1.080, *P* = 0.902) did not, compared to the rate of 100%. Urea reduction ratio (URR) was positively, while serum intact parathyroid hormone (iPTH), alkaline phosphatase (ALP), normalized protein catabolic rate (nPCR), and total phosphate-binding capacity of drug were negatively associated with achievement in target of serum phosphate.

**Conclusions:**

Keeping one-year achievement rate of serum phosphate higher than 50% provides significant clinical benefits in reducing cardiovascular mortality.

**Supplementary Information:**

The online version contains supplementary material available at 10.1186/s12882-021-02547-z.

## Introduction

Hyperphosphatemia is one of common complications in maintenance hemodialysis (MHD) patients which causes poor prognosis. Epidemiological investigations demonstrated that the prevalence of hyperphosphatemia in hemodialysis patients was 30–70% [1, 2] among different countries. It is obvious that serum phosphate level is far from well-controlled worldwide. This may be due to the exchange between serum phosphate and phosphate pool. Phosphate pool includes the phosphate which resides in extracellular compartments except for serum and bone [3]. Researchers believe that phosphate pool may reflect phosphate retention since it explains the rebound of serum phosphate level after hemodialysis treatment [4]. However, phosphate pool cannot be measured, and the association between phosphate pool and serum phosphate level remains uncertain. Thus, we propose that phosphate load, which contains phosphate pool and serum phosphate, is a biomarker for phosphate retention in hemodialysis patients.

Serum phosphate level is considered as an indicator for phosphate overload which is significantly associated with poor prognosis, especially the cardiovascular mortality [5–9]. However, single-time serum phosphate level seems not to be an ideal biomarker for phosphate overload. Dialysis Outcomes and Practice Patterns Study (DOPPS) found that increased serum phosphate level over 6.0 mg/dL was associated with increased all-cause mortality [10]. So as J Park, et al. [11] and H Wang, et al. [12] But K Kalantar-Zadeh, et al. [13] found serum phosphate concentration was not correlated with cardiovascular mortality in younger MHD patients. Recently, researchers have proposed other biomarkers besides serum phosphate level to assess phosphate overload. For instance, time-dependent serum phosphate (< 3.5 mg/dl or ≥ 7.0 mg/dL) [7] and time-averaged serum phosphate level (< 3.5 mg/dl or > 5.5 mg/dl) [13] are proved to be related with increased mortality. Unfortunately, the above biomarkers are a little confused and inconvenient to guide clinical practice. A practical method for phosphate load assessment is urgently needed.

Kidney Disease Outcomes Quality Initiative (KDIGO) Guideline [14] suggests lowering phosphate levels toward the normal range (2.5–4.5 mg/dL) in 2017 [15], and serum phosphate was recommended to be examined every 1 to 3 months for MHD patients. We proposed the conception “individual achievement rate of serum phosphate” which was defined as the proportion of serum phosphate levels within the target range (2.5–4.5 mg/dL) during a follow-up period for an individual patient. We supposed that “individual achievement rate of serum phosphate” would be more appropriate to reflect phosphate overload of a hemodialysis patient than other parameters of phosphate and was associated with the poor prognosis.

Thus, the primary aim of this study was to investigate the association of achievement rate of serum phosphate with cardiovascular mortality in hemodialysis patients. Besides, we explored the relative weights of related factors associated with achievement of serum phosphate in target range.

## Methods

### Study population

This was a single-center, retrospective cohort study. Patients receiving hemodialysis treatment for at least 3 months in Huashan Hospital from January 2012 to September 2019 were enrolled. Patients enrolled in January 2012 were divided into the previous group with exposure period from January 2012 to December 2012. Patients enrolled after January 2012 were divided into the new group with exposure period during the first year after enrollment. Exclusion criteria included age < 18 or > 85 years old, residual urine volume > 100 ml/d and missing data on key parameters including serum calcium, phosphate and iPTH. The patients flow chart was shown in Supplemental Table 1. A total of 336 patients on MHD were screened and 251 patients were finally enrolled in this study. The study was approved by the Ethics Committee on Human Research of Huashan Hospital, Fudan university (KY2021–584). Study design were shown in Supplemental Figure 2.

### Data collection, evaluation of individual achievement rate of serum calcium, phosphate and parathyroid hormone

Demographic, dialysis-specific and laboratory data were collected from electronic database of Hemodialysis Center. Patients in our center were followed regularly in January, April, July, and October with biochemical and immunological testing for the regular assessment of complications every year. The following laboratory parameters were determined: hemoglobin (Hb), serum calcium, phosphate, intact parathyroid hormone (iPTH), alkaline phosphatase (ALP), albumin (Alb), prealbumin (PAB), high-sensitivity C-reactive protein (hs-CRP) and N-terminal pro-B-type natriuretic peptide (NT-proBNP). The results of the first examination after enrollment within 3 months were considered as the baseline. The first year after enrollment was considered as the exposure period and the biochemical data during this period were included into analysis as predictors.

Blood samples were obtained at the beginning of dialysis on the day of the midweek HD session and all laboratory values were measured using Hitachi clinical chemistry analyzer (Tokyo, Japan) with standard laboratory techniques. The corrected calcium was calculated using the following formula [16]: Corrected Ca^2+^ (mmol/L) = Measured Ca^2+^ (mmol/L) + [40 - measured serum albumin (g/L)] × 0.02. The urea reduction ratio (URR) was calculated with the formula 100 × (1-[C_t_/C_0_]) [17], in which C_t_ was the blood urea nitrogen measured at the end of dialysis and C_0_ was the pre-dialysis blood urea nitrogen. The single-pool Kt/V delivered by HD (spKt/V_urea_) was estimated by the second-generation Daugirdas formula [18]. The normalized protein nitrogen appearance (nPNA) was calculated by the method described by Termorshuizen et al. [19] and normalized to standard body weight [20]. Total phosphate-binding capacity was defined as the product of dose of phosphate binders which a patient took per day and the amount of phosphate removal per tablet [21]. Meanwhile, the dose of calcitriol and the usage of cinacalcet were also recorded in the baseline and the routine follow-up.

According to the KDIGO guideline [14], the target level of serum calcium and phosphate were 2.10–2.60 mmol/L and 0.81–1.45 mmol/L respectively while the target level of iPTH was 150 to 300 pg/ml. The individual achievement rate of serum calcium, phosphate and iPTH was defined as the times of tests within the target range divided by total times of tests over a period of time. For instance, the calculation of one-year achievement rate of serum phosphate was the target-achieving times divided by the total four times in 1 year since we tested quarterly; long-term serum phosphate achievement rate was calculated by the target-achieving times divided by the number of tests in the whole follow-up period. The mean serum phosphate level and serum phosphate variability were also included in analysis. The mean serum phosphate level was defined as the average value of serum phosphate over 1 year or the whole follow-up period and serum phosphate variability was the coefficient of variation during the analysis period (CV, standard deviation/mean).

Cardiovascular mortality was regarded as the primary endpoint, defined as death due to acute myocardial infarction, pericarditis, cardiac tamponade, atherosclerotic heart disease, cardiomyopathy, cardiac arrhythmia, cardiac arrest, valvular heart disease, pulmonary edema, congestive heart failure or stroke. Enrolled patients were followed up from January 2012 to the day of censoring for death, transplantation, peritoneal dialysis, transferring to another center, or the end of the study (September 30th 2020).

### Statistical analyses

Quantitative variables were expressed as mean ± SD or as median (IQR), while categorical variables were expressed as percentages or ratios. The differences between groups were examined using χ2 test, independent t test or Kruskal-Wallis test appropriately. Crude hazard ratios (HR) for mortality was determined using univariable Cox regression model. Those parameters with *P* < 0.05 were selected to the multivariable stepwise Cox regression model to identify the independent risk factors and calculate the adjusted HR.

Random-intercept logistic mixed-effects models [22] were used to analyze the associations between achievement in target of serum phosphate (whether or not one patient meets the target of serum phosphate each time) and proposed risk factors. We included visit as a fixed categorical effect, and a random intercept for each subject in the model. Those parameters with *P* < 0.05 from the univariate analyses, in combination with the clinical relevance consideration, were selected to the multivariate logistic mixed-effects model [23]. Unadjusted and adjusted odds ratios (OR) as well as the 95% confidence interval (95% CI) were reported.

We explored the relative weights of related factors according to the standardized regression coefficients [24]. Logistic mixed-effects model which contained standardized variables was used to determine the relative weights of these factors. The standardized regression coefficients of the mixed-effects model were added as the total weight and the relative weights was calculated as the percentage of each variable in the total weight [25].

All analyses were performed using SPSS version 19 (Chicago, IL, USA) and Stata version 13 (College Station, TX, USA). A *p*-value of less than 0.05 was considered statistically significant.

## Results

### Baseline characteristics

A total of 251 hemodialysis patients from Huashan Hospital were enrolled in this study including 178 patients receiving hemodialysis before research (previous group) and 73 patients new to hemodialysis (new group). As Table 1 presented, the hemodialysis patients had a mean age of 61 ± 13 years old, 53.4% were male, 21.9% had diabetes mellites, and 34.7% had cardiovascular disease. Mean (±SD) serum calcium, phosphate and iPTH in the first year after enrollment were 2.45 ± 0.15 mmol/L, 1.77 ± 0.38 mmol/L and 309.5 ± 167.8 pg/ml, respectively. Patients in previous group had higher dialysis age [73.23(34.69,134.17) vs. 0 month, *P* < 0.001] and NT-proBNP [7081(3441,18,204) vs. 3744(2631,8483) pg/ml, *P* = 0.002] but lower serum albumin (38.85 ± 3.25 vs. 39.91 ± 3.54 g/L, *P* = 0.008). Meanwhile, the percentage of diabetes mellites was higher in patients new to hemodialysis (16.9% vs. 34.2%, P = 0.002). There were no significant differences of other characteristics between two groups.

**Table 1:** Demographic and clinical characteristics of hemodialysis patients

Characteristics	Overall(***N*** = 251)	Previous(***N*** = 178)	New(***N*** = 73)	***P***
**Age (year)**	61 ± 13	61 ± 12	59 ± 15	0.143
**Male (%)**	133(53.0%)	92(51.7%)	41(56.2%)	0.518
**Dialysis age (month)**	37.27(3.33,117.32)	73.23(34.69,134.17)	0	< 0.001
**Primary disease (%)**				0.173
Glomerulonephritis	87(34.7%)	63(35.4%)	24(32.9%)	
Diabetes	41(16.3%)	21(11.8%)	20(27.4%)	
Hypertension	39(15.5%)	29(16.3%)	10(13.7%)	
Polycystic kidney	27(10.8%)	20(11.2%)	7(9.6%)	
Other	57(22.7%)	45(25.3%)	12(16.4%)	
**Comorbidity**				
Diabetes mellitus	55(21.9%)	30(16.9%)	15(34.2%)	0.002
Cardiovascular disease	87(34.7%)	61(34.3%)	26(35.6%)	0.839
History of malignant tumor	26(10.4%)	19(10.7%)	7(9.6%)	0.798
**Laboratory test**				
Serum calcium (mmol/L)	2.45 ± 0.15	2.46 ± 0.15	2.41 ± 0.17	0.21
Serum phosphate (mmol/L)	1.77 ± 0.38	1.79 ± 0.37	1.72 ± 0.40	0.075
Serum iPTH (pg/ml)	309.5 ± 167.8	311.9 ± 170.7	280.2 ± 134.6	0.773
ALP (u/L)	96.42 ± 71.26	100.65 ± 64.49	92.11 ± 85.19	0.181
Hb (g/L)	107.78 ± 11.81	108.73 ± 11.44	105.49 ± 12.43	0.07
Alb (g/L)	39.16 ± 3.36	38.85 ± 3.25	39.91 ± 3.54	0.008
PAB (mg/L)	292.99 ± 74.15	284.97 ± 73.23	312.46 ± 73.22	0.071
hs-CRP (mg/L)	4.25 ± 3.19	4.45 ± 3.56	4.04 ± 3.13	0.34
NT-proBNP (pg/ml)	5797(3025,16,474)	7081(3441,18,204)	3744(2631,8483)	0.002
nPCR	1.13 ± 0.26	1.13 ± 0.25	1.12 ± 0.28	0.672
spKt/V	1.44 ± 0.29	1.46 ± 0.27	1.41 ± 0.31	0.169
URR	0.70 ± 0.08	0.70 ± 0.07	0.69 ± 0.09	0.29
Total Phosphate-binding capacity	88.23 ± 77.74	92.23 ± 58.42	83.91 ± 80.20	0.745
Calcitriol (ug/week)	0.75(0.00,1.88)	0.75(0.00,2.15)	0.75(0.00,1.25)	0.16
Cinacalcet	20(8.0%)	17(9.6%)	3(4.1%)	0.06

Values expressed as mean ± SD, median (IQR) or number(percentage); Previous group included patients received hemodialysis treatment before the research; New group included patients new to hemodialysis

Conversion factors for units: Alb and Hb in g/L to g/dL, ×0.1; calcium in mmol/L to mg/dL, ×4.0; phosphate in mmol/L to mg/dL, ×3.1; iPTH in pg/ml to pmol/L, ×0.11. No conversion is necessary for ALP, PAB, hs-CRP and NT-proBNP

*Abbreviations*: *iPTH* Intact parathyroid hormone, *ALP* Alkaline phosphatase, *Alb* Albumin, *Hb* Hemoglobin, *PAB* Prealbumin, *hs-CRP* High-sensitive C-reactive protein, *nPCR* Normalized protein catabolic rate, *URR* Urea reduction ratio

In 251 patients enrolled in our study, hypophosphatemia occurred in 53(21.1%) patients while hyperphosphatemia occurred in 243(96.8%) patients. The mean of individual one-year achievement rate of serum calcium, phosphate and iPTH were 68.7, 51.7 and 52.5%, respectively (Supplemental Figure 3). A total of 44 (17.5%) patients died from CVD during a median follow-up of 65 months.

### Serum phosphate achievement rate had higher predictive value than other parameters for cardiovascular mortality

**Fig. 1:**
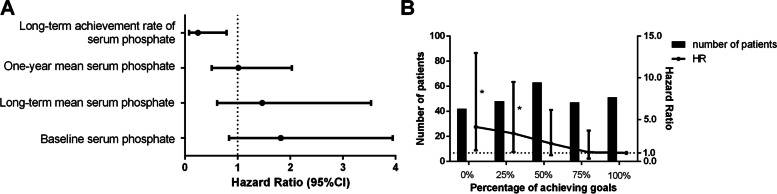
Association between serum phosphate indicators and CVD mortality in maintenance hemodialysis patients. Note: **A** Association between baseline, long-term and one-year serum phosphate indicators and CVD mortality by univariate Cox regression analysis. **B** Association between one-year achievement rate of serum phosphate and CVD mortality adjusted for age, diabetes mellitus, history of cardiovascular disease and one-year mean of serum albumin. HR, hazard ratio; 95%CI, 95% confidence interval. The calculation of one-year achievement rate of serum phosphate was the target-achieving times divided by the total four times in 1 year. * *P* < 0.05 compared to the group “One-year achievement rate of serum phosphate =100%”

In the unadjusted Cox model (Table 2), higher one-year achievement rate of serum phosphate was associated with lower risk of CVD mortality. Compared with one-year serum phosphate achievement rate of 100%, patients with rate of 0% (HR = 4.381, 95%CI = 1.179–17.283, *P* = 0.018) and 25% (HR = 3.244, 95%CI = 1.156–9.105, *P* = 0.025) had a higher risk of CVD mortality. Besides, higher long-term serum phosphate achievement rate (HR = 0.248, 95%CI = 0.078–0.791, *P* = 0.018) was also related to lower risk of CVD mortality (Fig. 1A, Supplemental Table 1). However, we did not find any association between CVD mortality and other parameters of calcium, phosphate and iPTH (Table 2).

**Table 2:** Predictive value of parameters of calcium, phosphate and iPTH for cardiovascular mortality by univariable Cox regression model

Parameters	CVD mortality		
	HR	*P*	95%CI
Baseline serum calcium	0.408	0.224	0.096–1.729
Baseline serum phosphate	1.820	0.129	0.840–3.945
Baseline serum iPTH	1.003	0.525	0.992–1.011
One-year mean serum calcium	0.910	0.892	0.236–3.512
One-year mean serum phosphate	1.015	0.965	0.507–2.032
One-year mean serum iPTH	1.001	0.158	0.998–1.002
One-year serum calcium variability	0.834	0.459	0.522–1.264
One-year serum phosphate variability	0.201	0.477	0.002–16.754
One-year serum iPTH variability	1.002	0.978	0.991–1.009
One-year achievement rate of serum calcium	–	–	–
Proportion = 0%	1.042	0.947	0.305–3.557
Proportion = 25%	1.961	0.116	0.846–4.545
Proportion = 50%	1.157	0.734	0.499–2.680
Proportion = 75%	1.115	0.799	0.481–2.586
Proportion = 100%	Ref	–	–
One-year achievement rate of serum iPTH	–	–	–
Proportion = 0%	1.554	0.394	0.563–4.288
Proportion = 25%	1.39	0.538	0.487–3.966
Proportion = 50%	1.507	0.362	0.624–3.638
Proportion = 75%	0.688	0.523	0.218–2.168
Proportion = 100%	Ref	–	–
One-year achievement rate of serum phosphate	–	–	–
Proportion = 0%	4.381	0.018	1.779–17.283
Proportion = 25%	3.244	0.025	1.156–9.105
Proportion = 50%	1.786	0.282	0.620–5.141
Proportion = 75%	1.494	0.493	0.474–4.708
Proportion = 100%	Ref	–	–

The calculation of one-year achievement rate of serum phosphate was the target-achieving times divided by the total four times in 1 year. The “Single” referred to the value of the baseline

*Abbreviation*: *iPTH* Intact parathyroid hormone

### Serum phosphate achievement rate was negatively associated with cardiovascular mortality

Univariate Cox analysis showed age, sex, diabetes mellitus, cardiovascular disease, history of kidney transplantation, one-year serum phosphate achievement rate, one-year mean of Alb, PAB, hs-CRP, NT-proBNP, spKt/V and URR were associated with cardiovascular mortality (Table 3). Stepwise multivariable Cox analysis further demonstrated that age (HR = 1.049, *P* = 0.005, 95%CI: 1.015–1.084), diabetes mellitus (HR = 1.979, *P* = 0.039, 95%CI: 1.036–3.778) and cardiovascular disease (HR = 2.790, *P* = 0.002, 95%CI: 1.468–5.303) increased the risk of cardiovascular mortality while lower Alb (HR = 0.848, *P* = 0.002, 95%CI: 0.762–0.943) were associated with higher CVD mortality. Besides, we found that hazard ratio for cardiovascular mortality was 4.117 (95%CI = 1.305–12.986, *P* = 0.016) in patients with one-year serum phosphate achievement rate of 0%, 3.343 (95%CI = 1.177–9.494, *P* = 0.023) in patients with rate of 25%, 2.129 (95%CI = 0.738–6.144, *P* = 0.162) in patients with rate of 50% and 1.080 (95%CI = 0.318–3.665, *P* = 0.902) in patients with rate of 75% compared to the reference group with rate of 100% (Table 3, Fig. 1B) Similar result was obtained for long-term serum phosphate achievement rate (Supplemental Table 2). Meanwhile, two additional adjustment models were performed since dialysis age and NT-proBNP were significantly different between previous group and new group (Supplemental Table 3). In both models, one-year achievement rate of serum phosphate was significantly associated with cardiovascular mortality.

**Table 3:** Cox regression analysis for the associations of various parameters and CVD mortality

Characteristics	Univariable		Multivariable		
	HR	*P*	HR	*P*	95%CI
Age	1.076	< 0.001	1.049	0.005	1.015–1.084
Male	2.053	0.026			
Dialysis age (month)	0.999	0.868			
Diabetes mellitus	2.773	0.001	1.979	0.039	1.036–3.778
Cardiovascular disease	3.463	< 0.001	2.79	0.002	1.468–5.303
History of malignant tumor	1.711	0.193			
History of kidney transplantation	0.258	0.045			
One-year achievement rate of serum phosphate	–	0.027	–	0.041	–
Proportion = 0%	4.381	0.012	4.117	0.016	1.305–12.986
Proportion = 25%	3.244	0.025	3.343	0.023	1.177–9.494
Proportion = 50%	1.786	0.282	2.129	0.162	0.738–6.144
Proportion = 75%	1.494	0.493	1.080	0.902	0.318–3.665
Proportion = 100%	–	Ref	–	Ref	–
One-year mean of Hb(g/L)	0.988	0.366			
One-year mean of Alb(g/L)	0.823	< 0.001	0.848	0.002	0.762–0.943
One-year mean of PAB(mg/L)	0.990	< 0.001			
One-year mean of hs-CRP(mg/L)	1.025	0.009			
One-year mean of NT-proBNP(pg/ml)	1.001	0.001			
One-year mean of nPCR	0.184	0.044			
One-year mean of spKt/V	0.113	0.009			
One-year mean of URR	0.001	0.003			

Parameters including one-year mean of ALP, Hb, Alb, PAB, hs-CRP, NT-proBNP, nPCR, spKt/V and URR were enrolled using the mean value of four examinations in a year. The calculation of one-year achievement rate of serum phosphate was the target-achieving times divided by the total four times in 1 year

*Abbreviations*: *iPTH* Intact parathyroid hormone, *ALP* Alkaline phosphatase, *Alb* Albumin, *Hb* Hemoglobin, *PAB* Prealbumin, *hs-CRP* High-sensitive C-reactive protein, *nPCR* Normalized protein catabolic rate, *URR* Urea reduction ratio

### ALP, iPTH, nPCR, URR, and total phosphate-binding capacity were related with target achievement of serum phosphate

**Fig. 2:**
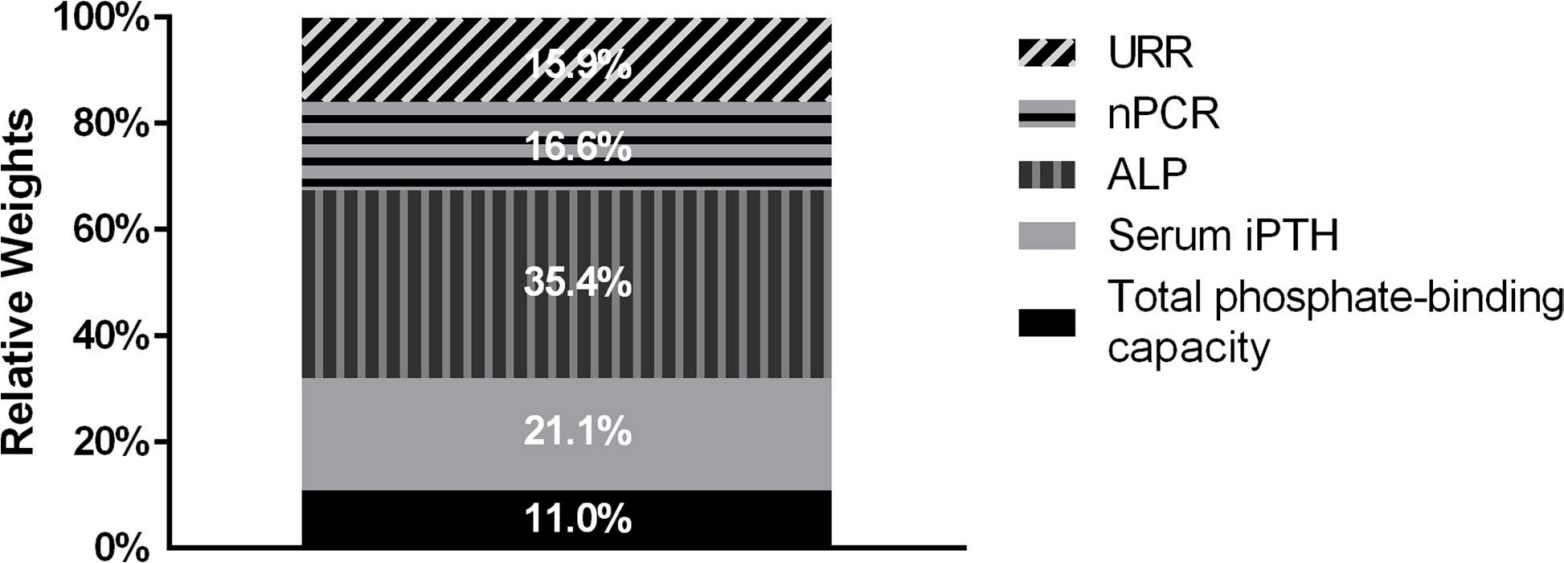
Relative weights of related factors for target achievement of serum phosphate in single time-point examination. Note: Abbreviations: iPTH, intact parathyroid hormone; ALP, alkaline phosphatase; nPCR, normalized protein catabolic rate; URR, urea reduction ratio

Logistic mixed-effects regression model was further used to explore the related factors of target achievement of serum phosphate during the first year. We found that serum iPTH level (HR = 0.922, *P* < 0.001, 95%CI: 0.903–0.950), ALP (HR = 0.957, *P* < 0.001, 95%CI: 0.967–0.983), nPCR (HR = 0.114, *P* < 0.001, 95%CI: 0.036–0.366), URR (HR = 2.259, *P* = 0.007, 95%CI: 2.393–3.924) and total phosphate-binding capacity of drug (HR = 0.997, *P* = 0.023, 95%CI: 0.995–0.999) were significantly correlated with target achievement of serum phosphate (Table 4). Relative weights analysis of related factors was presented in Fig. 2. Serum ALP level which contributed to 35.4% of the total weights had the greatest impact on target achievement of serum phosphate during the first year. Other parameters including iPTH, nPCR, URR, and total phosphate-binding capacity contributed to 21.1, 16.6, 15.9, and 11.0%, respectively.

**Table 4:** Multivariate mixed-effects logistic regression analysis of related factors for one-year achievement rate of serum phosphate

Characteristics	Univariable		Multivariable		
	OR	*P*	OR	*P*	95%CI
Age	1.026	0.015	1.162	0.219	0.991–1.040
Male sex	1.766	0.027	1.447	0.237	0.784–2.671
Dialysis age	1.002	0.174			
Serum Ca	2.054	0.088			
Serum iPTH	0.998	< 0.001	0.922	< 0.001	0.903–0.950
ALP	0.968	< 0.001	0.975	< 0.001	0.967–0.983
Alb	0.974	0.403			
PAB	0.996	0.069			
nPCR	0.139	0.001	0.114	< 0.001	0.036–0.366
spKt/V	4.265	0.005			
URR	5.202	< 0.001	2.598	0.007	2.393–3.924
NT-proBNP	1.001	0.138			
Total Phosphate-binding capacity	0.997	0.002	0.997	0.023	0.995–0.999
Calcitriol	0.955	0.017	1.001	0.972	0.959–1.045
Cinacalcet	1.459	0.252			

Parameters including ALP, Hb, Alb, PAB, hs-CRP, NT-proBNP, nPCR, spKt/V and URR are enrolled using the value of each visit during the first follow-up year. Dose of calcitriol and whether to use cinacalcet are recorded at each visit

Parameters including age, dialysis age, serum calcium, iPTH, ALP, Alb, PAB, nPCR, spKt/V, URR, NT-proBNP, total Pi-binding capacity, calcitriol are continuous variables while sex and cinacalcet are binary variables. The calculation of one-year achievement rate of serum phosphate was the target-achieving times divided by the total four times in 1 year

*Abbreviations*: *iPTH* Intact parathyroid hormone, *ALP* Alkaline phosphatase, *Alb* Albumin, *Hb* Hemoglobin, *PAB* Prealbumin, *hs-CRP* High-sensitive C-reactive protein, *nPCR* Normalized protein catabolic rate, *URR* Urea reduction ratio

## Discussion

In this retrospective analysis of 251 patients with maintenance hemodialysis, we found that individual achievement rate of serum phosphate had stronger predictive value for cardiovascular mortality than traditional calcium, phosphate and iPTH parameters. Hemodialysis patients with one-year target achievement rate of phosphate lower than 50% had higher cardiovascular mortality. Furthermore, serum iPTH level, ALP, nPCR, URR and total phosphate-binding capacity of drug were significantly correlated with target achievement of serum phosphate while serum ALP provided the largest contribution to target achievement of serum phosphate.

Single time-point measurement may not be an accurate biomarker of phosphate overload. Increased PTH, fibroblast growth factor 23 (FGF-23) and decreased Klotho, 1,25 dihydroxy vitamin D (1,25D) which regulate bone metabolism result in serum phosphate fluctuation [26, 27]. Moreover, Short-time high phosphate diets can alter the level of serum phosphate immediately [28, 29]. In addition, studies have found that the efficiency of phosphate binders varied from patients to patients. Thus, we propose that one-year achievement rate of serum phosphate instead of other parameters to reflect the status of phosphate metabolism in MHD patients accurately.

There are several reasons which may explain why one-year achievement rate of serum phosphate was appropriate to reflect phosphate metabolism. First, four times measurements are closer to the true state of phosphate control in patients. Second, one-year achievement rate of serum phosphate may indicate both serum phosphate level and serum phosphate variability. Serum phosphate level [30] and serum phosphate variability [31] are vital parameters for phosphate metabolism. If serum phosphate level fluctuates wildly during a period, serum phosphate is far from controlled although the time-averaged serum phosphate level may be normal. To some extent one-year achievement rate of serum phosphate can represent serum phosphate variability. Finally, the increased risk of cardiovascular mortality by phosphate was not a timely effect but a long-term effect through vascular calcification. One-year achievement rate of serum phosphate can be considered as the long-term effect. However, achievement rate of serum phosphate is an indirect indicator for phosphate load. New biomarkers for phosphate measurement should be explored in future study.

Since achievement rate of serum phosphate is associated with clinical outcome in hemodialysis patients, it is crucial for physicians to improve the achievement rate. In this study, we revealed that serum iPTH level, ALP, nPCR, URR and total phosphate-binding capacity of drug were main factors related with target achievement of serum phosphate. Among those factors, ALP had the largest relative weights while iPTH followed closely. As we know, ALP is generally considered as a nonspecific biomarker of bone metabolism [32]. S Salam, et al. [33] studied diagnostic accuracy of bone biomarkers, finding that total ALP could predict bone turnover with an area under curve (AUC) of 0.753. Other observational study also indicated that ALP was correlated with hungry bone syndrome [34] and chronic kidney disease-mineral metabolism disorder (CKD-MBD) [35]. Meanwhile, PTH plays a vital role in bone metabolism. S Salam, et al. [33] analyzed bone samples from 492 dialysis patients and demonstrated that iPTH was able to discriminate both low and high bone turnover from normal bone turnover. P Ivarsen et al. [36] reached the conclusion that PTH promoted bone resorption and was negatively associated with bone density. Basic research concluded similarly that elevated PTH led to reduced bone volume and increased bone turnover [37]. Thus, PTH may affect serum phosphate fluctuation through the impact on absorption and release of phosphate from bone. Our research found iPTH and ALP together occupied nearly 60% relative weights of target achievement of serum phosphate, illustrating that bone phosphate release contributed greatly to phosphate burden. Thus, to treat abnormal bone metabolism might be the major phosphate-lowing strategy. Another important factor is nPCR which reflects dietary protein intake [38]. Our results indicated nPCR occupied 16.6% relative weights for target achievement of serum phosphate, which meant restricted dietary phosphate intake was also essential for management of hyperphosphatemia. Similarly, URR and total phosphate-binding capacity of drug occupied 15.9 and 11.0% relative weights respectively, which indicated that dialysis adequacy and phosphate binders were useful for management of hyperphosphatemia as well. Though the above findings emphasize again the “3D” principles management of hyperphosphatemia, they also add strength to the order of treatment strategies.

In this study, we found that lower achievement rate of serum phosphate was an independent risk factor for cardiovascular mortality in maintenance hemodialysis patients. Therefore, it is urgent for physicians to take effective measures to improve such achievement rate. According to our results, we believe that keeping the one-year achievement rate of serum phosphate higher than 50% provides significant clinical benefits in reducing cardiovascular mortality. On one hand, compared with one-year serum phosphate achievement rate of 100%, the risk of cardiovascular mortality increased when achievement rate was 0 and 25% while it was not significantly different when achievement rate was 50 and 75%. On the other hand, the average one-year serum phosphate achievement rate of enrolled patients in this study was only 51.7%, which meant the achievement rate of many patients was less than 50%. It was difficult to raise one-year serum phosphate achievement rate up to 100% for all maintenance hemodialysis patients because of huge economic costs. Thus, we believe that it is an appropriate choice for physicians to keep the one-year achievement rate of serum phosphate higher than 50% of maintenance hemodialysis patients. This is also the major clinical value of this study.

Limitations to our study should be acknowledged. Firstly, due to the retrospective, observational nature of this study, we cannot prove causality between achievement rate of serum phosphate and CVD mortality. Secondly, our study was executed at single hemodialysis center in Huashan Hospital with a limited sample. Multicenter, prospective cohort study is needed. Finally, there may also be residual confounding or unmeasured confounders such as FGF-23 and vitamin D. In addition, we enrolled patients without residual renal function in this study because the residual renal function might influence the metabolism of calcium and phosphorus. Thus, the association of achievement rate of serum phosphate with CVD mortality in patients with residual renal function needs to be further investigated. Finally, hemodialysis patients with > 85 years old were rare in our hemodialysis center. Hence, it needs to be further investigated in old patients.

In conclusion, we demonstrate for the first time that achievement rate of serum phosphate is negatively correlated with cardiovascular mortality in maintenance hemodialysis patients. Keeping the one-year achievement rate of serum phosphate higher than 50% provides significant clinical benefits in reducing cardiovascular mortality. Achievement rate of serum phosphate is proved to be a more accurate indicator for phosphate load in maintenance hemodialysis patients, which is widely applicable for clinical practice.

## Supplementary Information

**Additional file 1: Supplemental Figure 1.** Patients Flow Chart. **Supplemental Figure 2.** Study design and follow-up. **Supplemental Figure 3.** Averaged One-year and long-term achievement rate of serum calcium, phosphate and iPTH. **Supplemental Table 1.** Comparison of long-term prognostic prediction value for CVD-cause mortality by univariable Cox regression model. **Supplemental Table 2.** Cox regression analysis for the association between long-term achievement rate of serum phosphate and CVD mortality. **Supplemental Table 3.** Adjusted models for the association between one-year achievement rate of serum phosphate and CVD mortality.

## Data Availability

The datasets used and/or analyzed during the current study are available from the corresponding author on reasonable request.

## Abbreviations

CVD: Cardiovascular disease
iPTH: Intact parathyroid hormone
ALP: Alkaline phosphatase
nPCR: Normalized protein catabolic rate
URR: Urea reduction ratio
MHD: Maintenance hemodialysis
DOPPS: Dialysis Outcomes and Practice Patterns Study
KDIGO: Kidney Disease Outcomes Quality Initiative
HB: Hemoglobin
Alb: Albumin
PAB: Prealbumin
hs-CRP: High-sensitivity C-reactive protein
NT-proBNP: N-terminal pro-B-type natriuretic peptide
FGF-23: Fibroblast growth factor 23
1,25D: 1,25 dihydroxy vitamin D
AUC: Area under curve
CKD-MBD: Chronic kidney disease-mineral metabolism disorder
