# Supplementary material for: Higher one-year achievement rate of serum phosphate associated with lower cardiovascular mortality in hemodialysis patients
Source: BMC Nephrol. 2021 Dec 1;22:398. doi: 10.1186/s12882-021-02547-z (PMC8638480; doi:10.1186/s12882-021-02547-z)
Supplement: Supplementary file 1 — Additional file 1: Supplemental Figure 1. Patients Flow Chart. Supplemental Figure 2. Study design and follow-up. Supplemental Figure 3. Averaged One-year and long-term achievement rate of serum calcium, phosphate and iPTH. Supplemental Table 1. Comparison of long-term prognostic prediction value for CVD-cause mortality by univariable Cox regression model. Supplemental Table 2. Cox regression analysis for the association between long-term achievement rate of serum phosphate and CVD mortality. Supplemental Table 3. Adjusted models for the association between one-year achievement rate of serum phosphate and CVD mortality. [file 12882_2021_2547_MOESM1_ESM.pdf]

Supplemental Figure 1. Patients Flow Chart

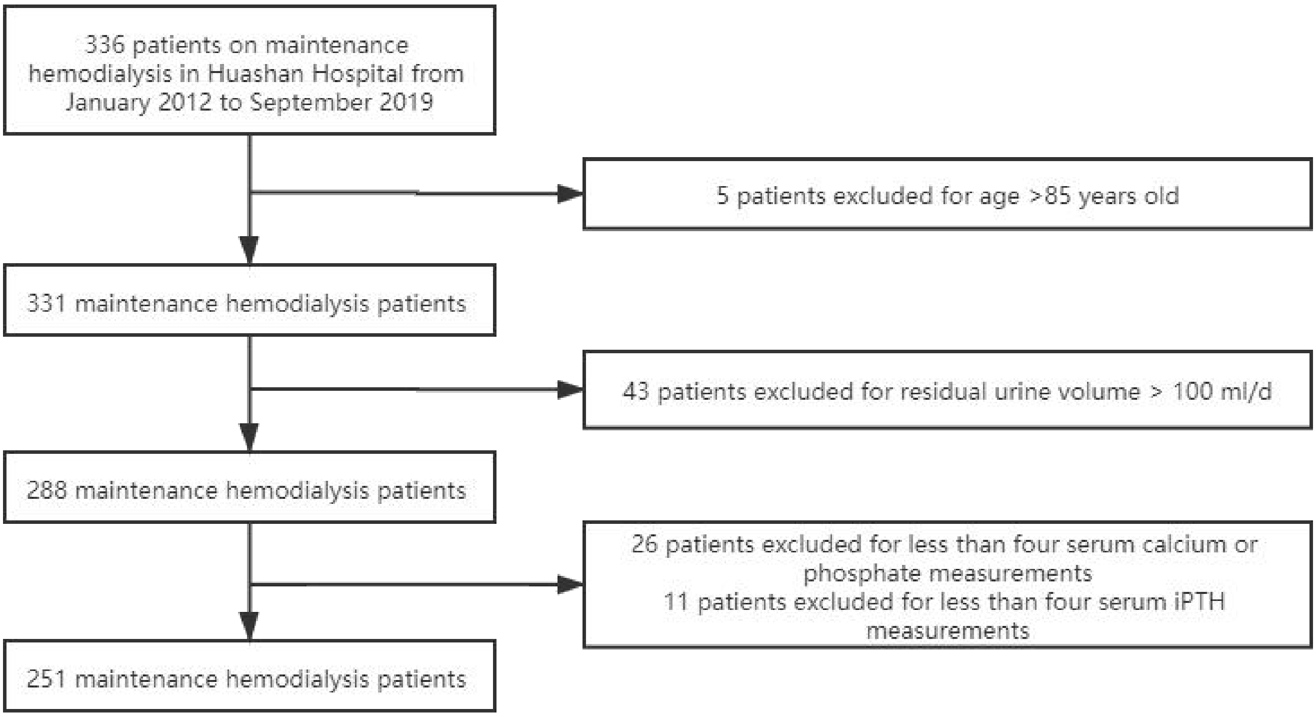

Supplemental Figure 2. Study design and follow-up

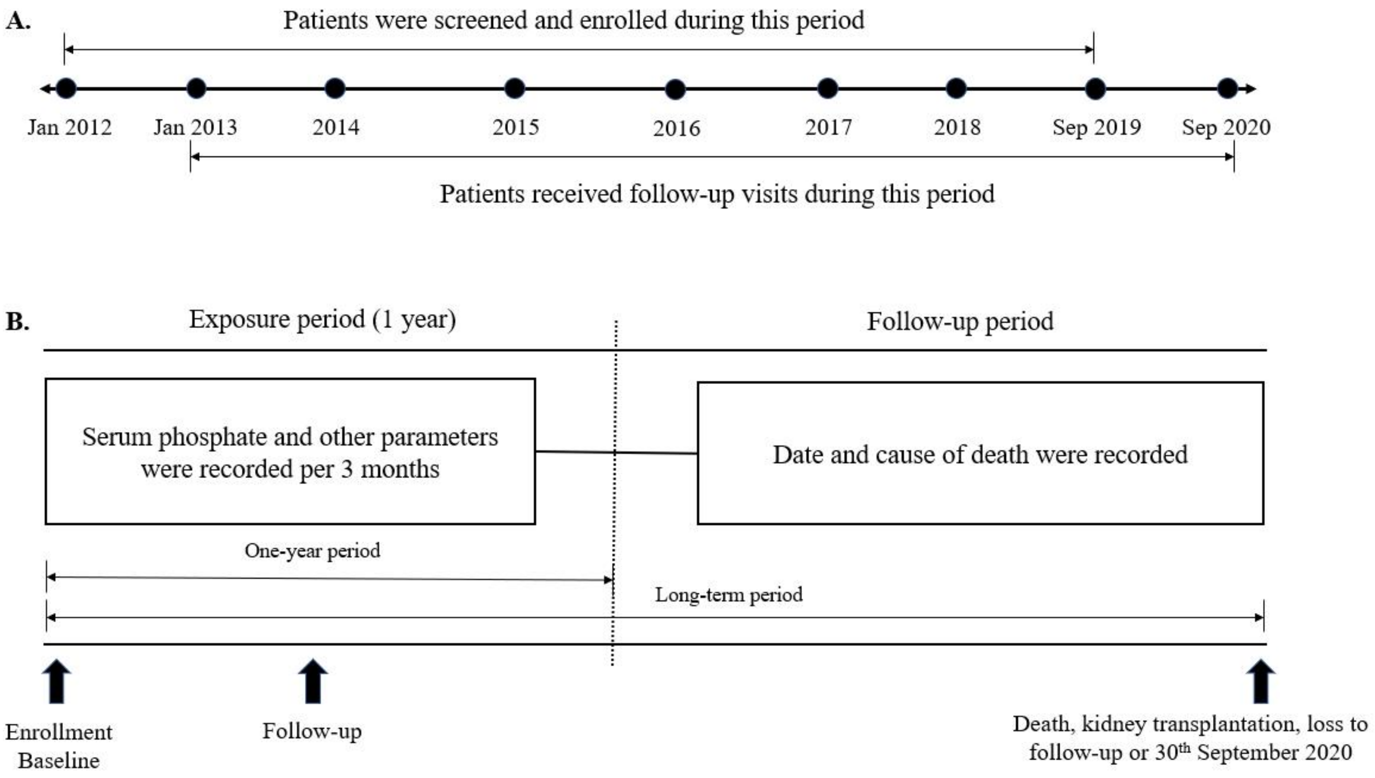

Note: Figure A showed the vintages during which patients were enrolled or received follow-up visits.

Figure B showed the exposure period and follow-up period. Patients who received maintenance hemodialysis from January 2012 to September 2019 were screened and enrolled. Those who were enrolled received follow-up visits until death, kidney transplantation, loss to follow-up or 30th September 2020.

Patients enrolled in January 2012 were divided into the previous group with exposure period from January 2012 to December 2012. Patients enrolled after January 2012 were divided into the new group with exposure period during the first year after enrollment.

Supplemental Figure 3. Averaged One-year and long-term achievement rate of serum calcium, phosphate and iPTH

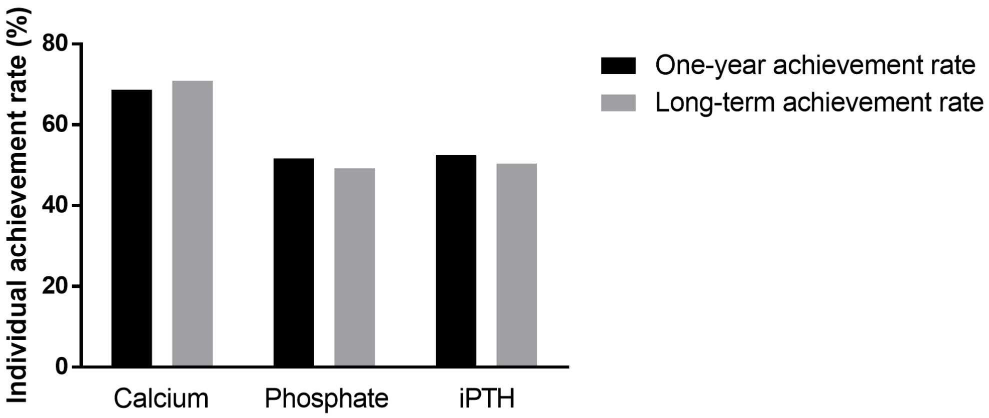

Supplemental Table 1. Comparison of long-term prognostic prediction value for CVD-cause mortality by univariable Cox regression model

Parameters	CVD-caused mortality		
	HR	P	95%CI
Long-term achievement rate of serum calcium	0.363	0.097	0.110-1.201
Long-term achievement rate of serum iPTH	0.482	0.24	0.143-1.627
Long-term mean serum phosphate	1.467	0.393	0.609-3.535
Long-term serum phosphate variability	0.092	0.304	0.001-8.690
Long-term achievement rate of serum phosphate	0.248	0.018	0.078-0.791

Note: Abbreviations: iPTH, intact parathyroid hormone;

The "Long-term" referred to the average value of the whole follow-up vintage.

Supplemental Table 2. Cox regression analysis for the association between long-term achievement rate of serum phosphate and CVD mortality

Characteristics	Univariable		Multivariable		
	HR	P	HR	P	95%CI
Age	1.076	<0.001	1.054	0.001	1.021-1.087
Male	2.053	0.026			
Dialysis vintage(month)	0.999	0.868			
Diabetes mellitus	2.773	0.001	2.546	0.004	1.343-4.827
Cardiovascular disease	3.463	<0.001	2.674	0.002	1.430-5.000
History of malignant tumor	1.711	0.193			
History of kidney transplantation	0.258	0.045			
Long-term achievement rate of serum phosphate	0.248	0.018	0.086	<0.001	0.025-0.303
Long-term mean of ALP(u/L)	1.008	<0.001			
Long-term mean of Hb(g/L)	0.959	0.009			
Long-term mean of Alb(g/L)	0.715	<0.001	0.759	<0.001	0.661-0.872
Long-term mean of PAB(mg/L)	0.992	0.005			
Long-term mean of hs-CRP(mg/L)	1.089	0.373			
Long-term mean of NT-proBNP(pg/ml)	1.001	0.001			
Long-term mean of nPCR	0.195	0.071			
Long-term mean of spKt/V	0.177	0.037			
Long-term mean of URR	0.003	0.047			

Note: Abbreviations: iPTH, intact parathyroid hormone; ALP, alkaline phosphatase; Alb, albumin; Hb, hemoglobin; PAB, prealbumin; hs-CRP, high-sensitive C-reactive protein; nPCR, normalized protein catabolic rate; URR, urea reduction ratio.

Parameters including long term mean of ALP, Hb, Alb, PAB, hs-CRP, NT-proBNP, nPCR, spKt/V and URR were enrolled using the mean value of the whole follow-up vintage.

Supplemental Table 3. Adjusted models for the association between one-year achievement rate of serum phosphate and CVD mortality

Characteristics	Original model			Adjusted model 1			Adjusted model 2		
	HR	P	95%CI	HR	P	95%CI	HR	P	95%CI
One-year achievement rate of serum phosphate	-	0.041	-	-	-	0.043	-	0.046	-
Proportion=0%	4.381	0.018	1.779-17.283	4.016	0.018	1.274-12.663	4.285	0.013	1.352-13.585
Proportion=25%	3.244	0.025	1.156-9.105	3.258	0.026	1.150-9.234	3.025	0.039	1.059-8.641
Proportion=50%	1.786	0.282	0.620-5.141	1.911	0.242	0.646-5.652	1.96	0.224	0.663-5.797
Proportion=75%	1.494	0.493	0.474-4.708	1.11	0.867	0.327-3.768	1.054	0.933	0.309-3.598
Proportion=100%	Ref	-	-	Ref	-	-	Ref	-	-

Note: Original model: Adjusted for age, diabetes mellitus, history of cardiovascular disease and serum albumin;

Adjusted model 1: Additional adjustment for dialysis age;

Adjusted model 2: Additional adjustment for serum dialysis age +NT-proBNP;
